# Development of a Smartphone App for a Genetics Website: The Amyotrophic Lateral Sclerosis Online Genetics Database (ALSoD)

**DOI:** 10.2196/mhealth.2706

**Published:** 2013-09-04

**Authors:** Olubunmi Abel, Aleksey Shatunov, Ashley R Jones, Peter M Andersen, John F Powell, Ammar Al-Chalabi

**Affiliations:** ^1^King's College LondonInstitute of PsychiatryDepartment of Clinical NeuroscienceLondonUnited Kingdom; ^2^Department of Pharmacology and Clinical NeuroscienceSection for NeurologyUmeå UniversitySE-901 85 UmeåSweden; ^3^King's College LondonDepartment of NeuroscienceLondonUnited Kingdom

**Keywords:** ALSoD, amyotrophic lateral sclerosis, frontotemporal dementia, Web-bases, database, genetics, bioinformatics, mobile website, app

## Abstract

**Background:**

The ALS Online Genetics Database (ALSoD) website holds mutation, geographical, and phenotype data on genes implicated in amyotrophic lateral sclerosis (ALS) and links to bioinformatics resources, publications, and tools for analysis. On average, there are 300 unique visits per day, suggesting a high demand from the research community. To enable wider access, we developed a mobile-friendly version of the website and a smartphone app.

**Objective:**

We sought to compare data traffic before and after implementation of a mobile version of the website to assess utility.

**Methods:**

We identified the most frequently viewed pages using Google Analytics and our in-house analytic monitoring. For these, we optimized the content layout of the screen, reduced image sizes, and summarized available information. We used the Microsoft .NET framework mobile detection property (HttpRequest.IsMobileDevice in the Request.Browser object in conjunction with HttpRequest.UserAgent), which returns a true value if the browser is a recognized mobile device. For app development, we used the Eclipse integrated development environment with Android plug-ins. We wrapped the mobile website version with the WebView object in Android. Simulators were downloaded to test and debug the applications.

**Results:**

The website automatically detects access from a mobile phone and redirects pages to fit the smaller screen. Because the amount of data stored on ALSoD is very large, the available information for display using smartphone access is deliberately restricted to improve usability. Visits to the website increased from 2231 to 2820, yielding a 26% increase from the pre-mobile to post-mobile period and an increase from 103 to 340 visits (230%) using mobile devices (including tablets). The smartphone app is currently available on BlackBerry and Android devices and will be available shortly on iOS as well.

**Conclusions:**

Further development of the ALSoD website has allowed access through smartphones and tablets, either through the website or directly through a mobile app, making genetic data stored on the database readily accessible to researchers and patients across multiple devices.

## Introduction

Amyotrophic lateral sclerosis (ALS) is a neurodegenerative disease of motor neurons resulting in progressive weakness of voluntary muscles. Death usually follows 2-5 years after the first symptoms appear, due to respiratory failure [1]. The causes of ALS are largely unknown, but a genetic component is present even in those without a family history of the disease. The gene variants contributing to risk have been identified in about 15% of the affected population, and the proportion is increasing rapidly. The ALS Online Genetics Database (ALSoD) [2] is a freely available database funded by major ALS charities (the ALS Association, the Motor Neurone Disease Association, ALS Canada, and MNDA Iceland) and sponsored by the European Network for the Cure of ALS and the World Federation of Neurology. It is aimed at researchers and clinicians and is designed for collation and bioinformatics analysis of ALS gene and phenotypic information. It typically receives about 300 unique visits a day [3,4].

Like most websites, ALSoD was initially built to display information to users of desktops and laptops, and the pages are configured to suit the height and width of those screens. With changes in browsing habits, website access is now often through a small portable device like a smartphone or tablet computer. It is therefore essential to ensure data will display correctly on a small device [5,6]. Mobile device data traffic has overtaken desktop traffic in the last decade, and data traffic on mobile devices for browsing alone has risen more than four times in 2008 [5]. According to NetMarketShare, the introduction of the Apple mobile device operating system, iOS, for the Apple iPad and iPhone for mobile browsing between March and October 2010 doubled Internet traffic [7], leading to a projection that, by 2014, mobile Internet usage will overtake desktop Internet usage [8]. Although the target community for ALSoD is mainly university or hospital-based users where desktop and laptop computers are common, such users are increasingly likely to use a portable device for use in clinic settings, conferences, or the laboratory, where fast access away from an office may be needed. Thus, it is essential that the ALSoD website is accessible not just from a desktop or laptop computer, but also from portable devices.

Mobile phone displays have greatly improved from the early monochrome screens for sending SMS messages to colorful graphical touch screens for mobile browsing [9]. Issues of low bandwidth and low resolution screens have been resolved, and smartphones and tablets should be regarded as “mobile computers” [10-13]. It therefore makes sense to write webpages specifically for portable devices. Mobile webpage content is similar to desktop webpage content and uses HTML connected and accessible over the Internet, even though mobile websites are typically accessed through Wi-Fi, 3G, or 4G networks [14]. Furthermore, portable devices are able to use applications (apps) specific to a website for access, rather than a generic browser, with the advantage of offline access to some information. Apps are generally platform-specific and downloaded from company portals, for example, BlackBerry App World, Apple App Store, and Google Play [14]. Thus, as well as writing ALSoD webpages specific for mobile devices, we also aimed to design a platform-specific app to enable some offline content and improve the user experience.

## Methods

### Optimization of Webpages

Our first focus was on the development of a mobile Web-based platform because we wanted the content to work across all mobile platforms [6]. Identification of the most viewed information was carried out using in-house analytic data coupled with the Google Analytics service configured for the ALSoD website. The Google Analytics tool was configured in August 2012. We based our data analysis on the 3 subsequent months (August, September, and October). These represent the period from when the Google Analytics tool was implemented to the point where we started the development of the mobile website and will be referred to as the pre-mobile website period. The 3-month period from November 2012 to January 2013 with the mobile website fully developed and the app implemented will be referred to as the post-mobile website period. In the pre-mobile website period, page views (the total number of pages viewed) were analyzed to discover the commonly visited pages on the website, including repeated views of a single page.

### Design Heuristics

Designing a mobile website that works on several platforms does not mean shrinking a complete webpage into a mini-sized webpage; the aim is to eliminate very small type, scrolling left or right, and typing, and to achieve the outcome required with a single click [6,15]. We therefore created a separate style sheet, retained some original images, reduced the size of images by a fixed percentage, configured the content layout of the screen to wrap text, and summarized the information on the desktop version to fit a smaller screen.

### Mobile Device Detection

**Figure 1:**
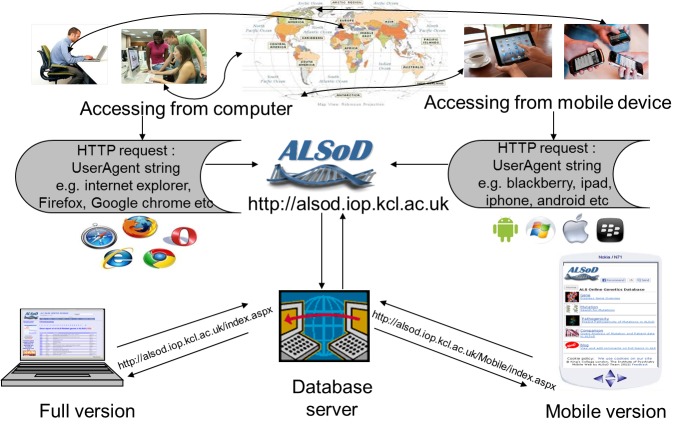
Overview of mobile website development.

To detect if the request comes from a mobile device, the .NET Framework provides the “isMobileDevice” property, which returns a true value if the browser is a recognized mobile device. This does not always work because some mobile device browsers disguise themselves as desktop browsers [16]. We therefore use “UserAgent” strings sent by the mobile device browser to the server in conjunction with the “isMobileDevice”, as described in Multimedia Appendix 1 and with an overview in Figure 1.

### Requesting Responses

**Figure 2:**
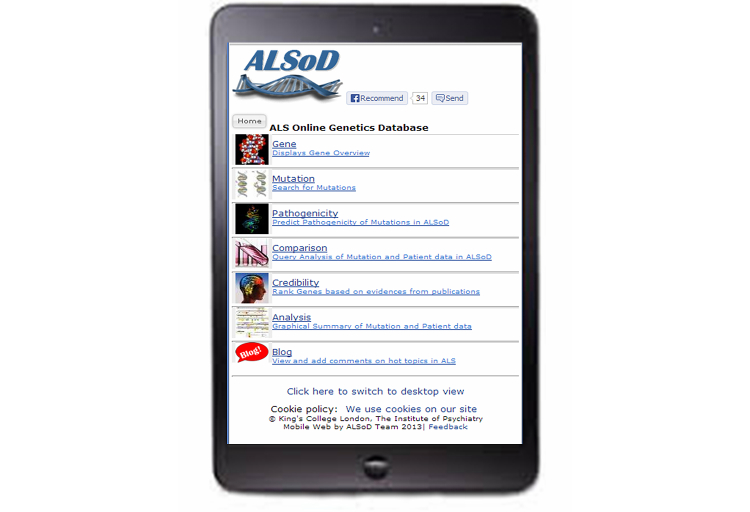
Mobile view of website.

Text messages and BlackBerry Messenger messages were sent to a selection of individuals known to the authors, asking them to view the ALSoD Web address on their phones and tablets. After the app was developed, additional users were asked to download the app via Google Play. Since ALSoD has a Facebook [17] account, a Facebook “Recommend” Button was embedded on the mobile master page. All users were asked to click on the Facebook Recommend button so as to have an estimate of the number of users who were satisfied with the outcome of the display on their phone, as seen in Figure 2.

### App Development

The most commonly used smartphone platforms are iOS, BlackBerry, Android, and Windows Mobile. We used Eclipse software as the integrated development environment (IDE), with the Android software development kit (SDK), Android development tools (ADT) plugin, BlackBerry plugin, and the latest SDK tools and platforms, downloaded using the SDK Manager [18-22].

### App Submission

From Eclipse, the application was compiled producing an .apk file (Android application package file format). This file was submitted to a registered Google Play account with a generated keystore containing a private key [23]. The ALSoD app can be downloaded from Google Play and currently, our Google app account confirms that the ALSoD app has had more than 100 downloads. The app then displays the website (designed using the Microsoft ASP.NET framework) with no status bar or URL navigation on the screen.

### Creating Awareness

**Figure 3:**
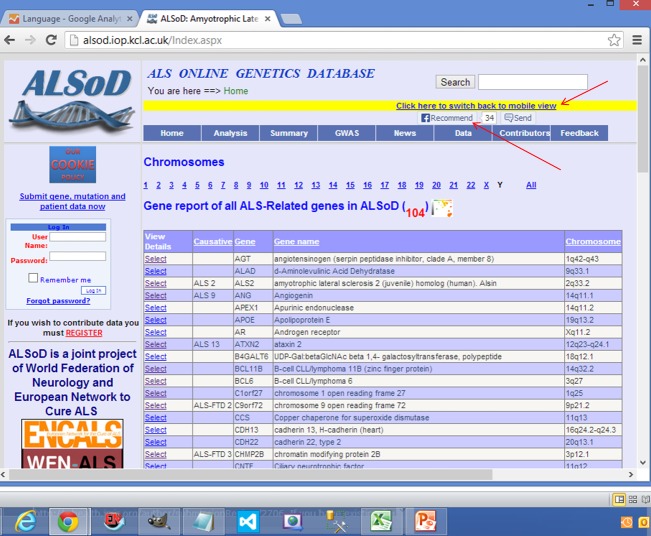
Desktop view creating awareness using Facebook and a marquee.

A marquee function scrolling text from right to left was inserted on the desktop master page to create an alert for regular users of the website, as seen in Figure 3. At symposiums and seminars, researchers were exposed to the recent development of the mobile app, which has contributed to increased Web traffic to ALSoD.

### Feedback From App Users

During the various presentations of the mobile app development through posters and seminars, practical assessment of the website on mobile phones was carried out by attendants. Responses, questions asked, concerns raised, and critical analysis given by the audience were recorded and considered.

### Analysis of Visits

The Google Analytics account for ALSoD was created in August 2012 to compare results generated by the two tools (mobile website and app) and to gain insight into the changes of the design and content of the website [24]. Visits were compared for the period before mobile website development, from August 2012 to October 2012, with the period after, from November 2012 to January 2013.

## Results

### Optimization of Webpages

In the 6-month data analysis period from August 2012 to January 2013, there were 5051 visits to the website, of which 2698 were unique (53%). There were 19,785 page views, of which 8883 (45%) were to 4 sets of pages. These pages focused on the pathogenicity of mutations, gene information, data analysis of mutations, and patient data.

### Design Heuristics

Pages were optimized for mobile browsing by reducing image size to 5% of the original size, creating a mobile master page different from the desktop master page, and creating a link page to allow users to switch seamlessly between the mobile and desktop views.

### Mobile Device Detection

If the UserAgent string contained keywords suggesting a mobile platform, for example, BlackBerry, Palm, mobile, iPhone, or iPad, then the user’s device was redirected to the mobile site [25] displaying the compact version (Figure 2) of the website instead of the full version (Figure 3) [11].

### Requesting Responses

To test this, we sent the mobile site URL to mobile phones of 14 users (colleagues and friends from whom we could easily obtain verbal feedback): 3 on the Android platform, 1 on the Windows Phone, 5 on BlackBerry OS, and 5 on iOS (2 iPhone and 3 iPad). All users gave positive feedback except for the Windows Phone user who could not utilize the pages with dropdown boxes.

### App Development

Following successful implementation of the mobile website, we began app development. One straightforward method to achieve this is to automatically convert an already-built mobile website into a native app. This is done through the “WebView” object, which is an in-app Web browser used to display a website as if viewed on the browser of an Android smartphone [11]. For testing, we downloaded and used Android simulators. The plugins allow programmers to develop, test, and debug a Java application using the Eclipse IDE, but it requires a high level of programming skill [26]. We also tested and manipulated the .apk file on a real Android phone before submitting to Google Play.

### Creating Awareness

**Figure 4:**
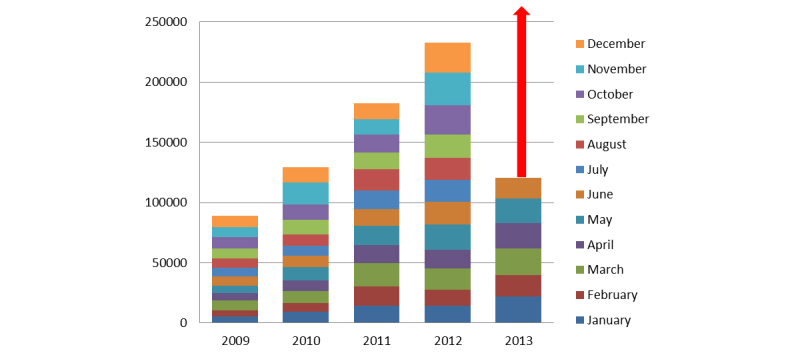
Graphical display of increased Web traffic on ALSoD.

Following the development of the mobile version, on the ALSoD Facebook page, 34 users recommended the website by May 29, 2013, using the Facebook “Recommend” button embedded on the website. Current tabular data are available on the website [27], displaying the growth of visits to the genetic database, as seen graphically in Figure 4.

### Feedback From App Users

After the creation, testing, and publicity of the app, we received feedback from users about: caching for offline viewing [28-30], which would enable users to continue work; having a “page loading” icon when connecting; making users aware of the cookies policy; using an option menu button [31] to display analysis webpages (interaction.aspx, credibility.aspx, analysis.aspx); and creating a link to allow users to switch from mobile view to desktop view, as this would be useful on tablets like the iPad. We were able to implement all changes except for the offline viewing, which is difficult to implement because the database is large and held online.

### Analysis of Visits

Our Google Analytics account showed that visits to the website increased from 2231 to 2820, yielding a 26% increase from pre-mobile period to post-mobile period and a 230% increase on the use of mobile devices (including tablets) to access the ALSoD website. On average, there were 300 unique visitors a day suggesting a high demand from the research community. A total of 1595 unique visitors in the post-mobile era accessed 11,376 page views on the website as opposed to 1220 unique visitors in the pre-mobile period (an increase of 31%), showing the relevance of a mobile-friendly website. Five mobile operating systems (Android, iOS, BlackBerry, Windows Phone, Symbian) were detected to have accessed the website within 6 months. Although BlackBerry OS visits declined from 34 to 14 visits (58%), iOS for iPhones and iPads increased from 40 to 105 visits (162%), and visits by Android devices increased from 29 to 213 visits (634%) (see Table 1). The Google search engine was the most used to search for the website (see Table 2). The likely explanation for the great increase in the use of Android devices is the development and introduction of the Android app submitted to Google Play.

**Table 1:** Comparison of website visits between the pre-mobile and post-mobile development.

Operating system		Visits	Pages per visit	Avg visit duration	% new visits	Bounce rate, %
**Totals**						
		26.40%	7.03%	1.48%	4.25	2.98
		2820 vs 2231	4.03 vs 3.77	00:03:55 vs 00:03:58	52.45 vs 54.77	47.45 vs 48.90
**Windows**						
	01/Nov/2012-31/Jan/2013	1945	4.25	00:04:20	49.56	44.78
	01/Aug/2012-31/Oct/2012	1564	3.85	00:04:13	56.46	48.34
	% Change	24.36	10.47	2.71	-12.21	-7.36
**Macintosh**						
	01/Nov/2012-31/Jan/2013	435	3.26	00:02:36	51.26	53.33
	01/Aug/2012-31/Oct/2012	490	3.27	00:02:50	51.02	54.08
	% Change	-11.22	-0.28	-8.71	0.48	-1.38
**Android**						
	01/Nov/2012-31/Jan/2013	213	2.35	00:01:29	76.53	63.85
	01/Aug/2012-31/Oct/2012	29	4.93	00:03:12	65.52	44.83
	% Change	634.48	-52.30	-53.69	16.80	42.43
**iOS**						
	01/Nov/2012-31/Jan/2013	105	4.52	00:02:31	82.86	48.57
	01/Aug/2012-31/Oct/2012	40	4.55	00:02:06	87.50	50.00
	% Change	162.50	-0.58	19.77	-5.31	-2.86
**Linux**						
	01/Nov/2012-31/Jan/2013	85	6.51	00:08:11	28.24	29.41
	01/Aug/2012-31/Oct/2012	61	3.23	00:03:13	34.43	32.79
	% Change	39.34	101.45	154.08	-17.98	-10.29
**Other systems**						
	01/Nov/2012-31/Jan/2013	15	1.07	00:00:28	100.00	93.33
	01/Aug/2012-31/Oct/2012	13	1.92	00:00:43	92.31	84.62
	% Change	15.38	-44.53	-35.61	8.33	10.30
**BlackBerry**						
	01/Nov/2012-31/Jan/2013	14	8.14	00:11:10	0.00	28.57
	01/Aug/2012-31/Oct/2012	34	7.12	00:14:12	5.88	17.65
	% Change	-58.82	14.40	-21.31	-100.00	61.90
**Windows Phone**						
	01/Nov/2012-31/Jan/2013	4	6.75	00:08:02	50.00	50.00
	01/Aug/2012-31/Oct/2012	0	0	00:00:00	0.00	0.00
	% Change	∞	∞	∞	∞	∞
**LG**						
	01/Nov/2012-31/Jan/2013	3	1.33	00:00:11	0.00	66.67
	01/Aug/2012-31/Oct/2012	0	0	00:00:00	0.00	0.00
	% Change	∞	∞	∞	0.00	∞
**Samsung**						
	01/Nov/2012-31/Jan/2013	1	1	00:00:00	100.00	100.00
	01/Aug/2012-31/Oct/2012	0	0	00:00:00	0.00	0.00
	% Change	∞	∞	0.00	∞	∞

**Table 2:** Referral traffic from search engines from August 2012 to June 2013.

Source	Visits	Pages per visit	Avg. visit duration	% new visits	Bounce rate, %
Google	4339	4.06	00:04:54	42.73	46.58
Yahoo	62	3.63	00:02:42	51.61	41.94
Bing	32	5.53	00:07:11	62.50	31.25
Baidu	21	6.19	00:12:50	23.81	23.81
Daum	13	3.46	00:06:35	30.77	30.77
Ask	10	4.9	00:08:33	20.00	20.00
Conduit	8	17.62	00:08:27	37.50	25.00
AOL	7	1	00:00:00	71.43	100.00
Other search engines	5	6	00:04:24	0.00	0.00
Yandex	4	1.25	00:00:04	100.00	75.00
Babylon	3	1.33	00:00:33	100.00	66.67
AVG	1	1	00:00:00	100.00	100.00
Comcast	1	1	00:00:00	100.00	100.00

## Discussion

### Principal Findings

ALSoD was developed as a disease-specific database for ALS, focused on genetics and phenotype, with planned incorporation of environmental and other risk factors in the future. We have shown that development of the website to facilitate smartphone access has greatly increased access.

In broad terms, there are two strategies for development of an app like this. Either the app can be developed as stand-alone software or it can be developed as a means to access an existing mobile website. We chose access to a mobile website for several reasons. First, we used this approach because mobile websites are immediately accessible to users through a browser, more compatible across devices, have easier content updates, are faster to find on search engines, make it easier to share content via a link, have a longer lifecycle on a user’s device, are easily convertible to an app, and are more cost-effective [14,32]. Second, the database is regularly updated with data, which would require the release of weekly updates to an app if the website was not the primary content holder. Third, although third-party automated app development tools exist [33,34], it was simple for us to convert the mobile website into an Android app using the WebView object.

A limitation to this approach is that the website recognizes a mobile device based on the information held in the list in the UserAgents string. Although software exists to automatically update the list, there is a financial cost involved and we have therefore chosen to update the list manually. Furthermore, UserAgents strings are limited in the kind of information sent to the server. Specific information such as the size of the screen, the manufacturer, the format of image supported, and the model of phone are not sent. This is an issue because some mobile devices allow portrait and landscape views when repositioned while others have unique width and height dimensions. It is therefore difficult to have a perfect display on all devices.

There are 3 main methods by which users access the website (Table 2). Roughly 39% of the traffic is organic from the Google search engine, 37% is direct by typing the ALSoD URL directly on a browser, and the rest are referrals through external sites collaborating with ALSoD.

More than 100 interactive, downloadable widgets and mobile applications have been submitted to the NHS Choices Health Apps library [35]. Some of these apps are commercial apps and the freely available ones range from calculating alcohol consumption to weight tracking. There are no specific genetics disease apps that concentrate on combining genotype, phenotype, and geographical information with associated analysis tools, although ALS database apps do exist. For example, the PatientsLikeMe app was initially developed to help United Kingdom-based ALS patients find clinical trials that are right for them and organizations find patients right for their trials [36,37]. The ALSoD app has direct relevance to clinicians working in ALS and therefore relevance to the NHS Choices Health Apps library. It includes a comparison tool to evaluate information for different genes side by side or jointly with user configurable features, a pathogenicity prediction tool using a combination of computational approaches to distinguish variants with nonfunctional characteristics from disease-associated mutations with more dangerous consequences, and a credibility tool to enable ALS clinicians and researchers to objectively assess the evidence for gene-causation in ALS. A checklist, as seen in Multimedia Appendix 2, was used to report the web-based intervention of users [38]. Furthermore, integration of external tools, systems for feedback, annotation by users, and two-way links to collaborators hosting complementary databases further enhance the functionality.

### Conclusions

Development of the mobile website and associated app has increased access to this disease-specific database and facilitated access through a wide range of devices. Visitor analysis has shown the importance of collaborating with other relevant databases through hyperlinks. Our future work will concentrate on further integration with other databases, adding in nongenetic risk factors, and increasing access and relevance for related research disciplines.

## Multimedia Appendix 1

Script for redirecting views.

## Multimedia Appendix 2

CONSORT-EHEALTH checklist V1.6.2 [38].

## Abbreviations

ALSoD: Amyotrophic Lateral Sclerosis Online Genetics Database
ASP.NET: active server page network
IDE: integrated development environment
Master page: provides automatic layout, pagination, headers and footers, and graphic elements for multiple pages.
OS: operating system
SMS: short message service
UserAgent: a user agent is software (a software agent) that is acting on behalf of a user.
Wi-Fi: wireless local area network
