# Supplementary material for: Development of a Smartphone App for a Genetics Website: The Amyotrophic Lateral Sclerosis Online Genetics Database (ALSoD)
Source: JMIR Mhealth Uhealth. 2013 Sep 4;1(2):e18. doi: 10.2196/mhealth.2706 (PMC4114449; doi:10.2196/mhealth.2706)
Supplement: Supplementary file 1 [file mhealth_v1i2e18_app1.pdf]

```
//Script for redirecting users from desktop to mobile view
//Programmer: Olubunmi Abel
//Date: 31st July 2013

<script language="C#" runat="server">

protected void Page_Load(object sender, EventArgs e)
    {

        DetectUserAgent();
        DataView dv1 =
(DataView)SqlDataSource2.Select(DataSourceSelectArguments.Empty);
        Label1.Text = dv1.Table.Rows[0][0].ToString();
    }

        void DetectUserAgent()
        {
                string strUserAgent = Request.UserAgent.ToString().ToLower();
        if (strUserAgent != null)
                {
        if (Request.Browser.IsMobileDevice == true ||
((strUserAgent.Contains("iphone") || strUserAgent.Contains("ipad") ||
strUserAgent.Contains("blackberry") || strUserAgent.Contains("mobile") ||
strUserAgent.Contains("windows ce") || strUserAgent.Contains("opera mini") ||
strUserAgent.Contains("palm") || strUserAgent.Contains("android") ||
strUserAgent.Contains("samsung") || strUserAgent.Contains("nokia"))))
                {
                        Response.Redirect("~/Mobile/index.aspx");
                }
                }
        }

</script>
```
