# Supplementary material for: Development of a Smartphone App for a Genetics Website: The Amyotrophic Lateral Sclerosis Online Genetics Database (ALSoD)
Source: JMIR Mhealth Uhealth. 2013 Sep 4;1(2):e18. doi: 10.2196/mhealth.2706 (PMC4114449; doi:10.2196/mhealth.2706)
Supplement: Supplementary file 2 [file mhealth_v1i2e18_app2.pdf]

CONSORT-EHEALTH Checklist V1.6.2 Report

(based on CONSORT-EHEALTH V1.6), available at [http://tinyurl.com/consort-ehealth-v1-6].

2706

Date completed

6/20/2013 20:26:25

by

Olubunmi Abel

Development of a smartphone app for a genetics website: The ALS Online Database (ALSoD)

TITLE

1a-i) Identify the mode of delivery in the title

"smartphone app "

1a-ii) Non-web-based components or important co-interventions in title

NA

1a-iii) Primary condition or target group in the title

"ALS"

ABSTRACT

1b-i) Key features/functionalities/components of the intervention and comparator in the METHODS section of the ABSTRACT

"Feedback from 14 testers informed our decision on developing an Android app. For app development, we used the Eclipse integrated development environment with Android plug-ins. We wrapped the mobile website version with the WebView object in Android. Simulators were downloaded to test and debug the applications. We then compared visits from the pre-mobile website period (August 2012 to October 2012) with visits from the post-mobile website period (November 2012 to January 2013) and a Facebook 'Recommend' Button embedded on the website is voluntarily clicked by the user to confirm their satisfaction with the new development."

1b-ii) Level of human involvement in the METHODS section of the ABSTRACT

"We used the .NET framework mobile detection property which returns a true value if the browser is a recognized mobile device and redirects users automatically"

1b-iii) Open vs. closed, web-based (self-assessment) vs. face-to-face assessments in the METHODS section of the ABSTRACT

"To test this, we sent the mobile site URL to mobile phones of 14 users (colleagues and friends from whom we could easily obtain verbal feedback), three on the Android platform, one Windows Phone, five Blackberry OS, five iOS – two iPhone and three iPad."

1b-iv) RESULTS section in abstract must contain use data

"From our Google Analytics account, visits to the website increased from 2,231 to 2,820 yielding a 26% increase from pre-mobile period to post-mobile period and a 230% (from 103 to 340 visits) increase on the use of mobile devices (including tablets) to access the ALSoD website. "

1b-v) CONCLUSIONS/DISCUSSION in abstract for negative trials

"Statistics here show that apart from the huge impact a search engine has on a database, collaboration with other databases has a role to play in the exploration of valuable resources available on a website. Also, the increased use of the website due to its mobile-friendliness has been analysed using Google analytics tool showing that more researchers are using mobile devices to enhance their research work globally."

INTRODUCTION

2a-i) Problem and the type of system/solution

"Amyotrophic lateral sclerosis (ALS) is a neurodegenerative disease of motor neurons killing patients within 2-5 years of diagnosis due to failure of the respiratory system [1]. With increasing growth of data generated by researchers in the field of ALS, a freely available database that has been transformed from a single gene storage facility recording mutations in the SOD1 gene to a multigene ALS bioinformatics repository and analytical instrument combining genotype, phenotype, and geographical information with associated analysis tools was developed [2, 3]. The website, the ALS Online Genetics Database, ALSoD (http://alsod.iop.kcl.ac.uk) is accessed mainly by the ALS research or clinical community, and typically receives about 300 unique visits per day. Its usage is commonly by researchers who require up-to-date genetic materials online, clinicians who want more information about patient data and patients or their families who desire geographical data about their specific mutation."

2a-ii) Scientific background, rationale: What is known about the (type of) system

"Like most websites, ALSoD was initially built to display information for users of desktops and laptops, and the pages are configured to suit the height and width of those screens. With changes in browsing habits, website access is often through a small portable device like a smartphone or tablet computer. It is therefore essential to ensure data will display correctly on a small device [4, 5]. Mobile device data traffic has overtaken desktop traffic in the last decade, and data traffic on mobile devices for browsing alone has risen over four times in 2008 [4]. According to Netwmarketshare, the introduction of the Apple mobile device operating system, iOS, doubled the use of the Apple iPad and iPhone for mobile browsing between March and October 2010 [6], leading to a projection that by 2014 mobile internet usage should overtake desktop internet usage [7]. Although the target community for ALSoD is mainly university or hospital based where desktop and laptop computers are common, such users are increasingly likely to use a portable device for use in clinic settings, conferences or the laboratory, where fast access away from an office may be needed. Thus, it is essential that the ALSoD website is accessible not just from a desktop or laptop computer, but also from portable devices."

METHODS

3a) CONSORT: Description of trial design (such as parallel, factorial) including allocation ratio

"By developing a mobile-friendly website and app, we sought to compare data traffic within 6 months to authenticate the significance of ALSoD to the ALS research community internationally."

3b) CONSORT: Important changes to methods after trial commencement (such as eligibility criteria), with reasons

"These first 3 months represented the period from when the Google Analytics tool was implemented to the point where we started the development of the mobile website (which will be referred to as the pre-mobile website period). The post-mobile website period is the second 3 months from November 2012 to January 2013 where the mobile website has been fully developed and an app implemented. In the pre-mobile website period, Pageviews (which is the total number of pages viewed) were analysed to discover the commonly visited pages on the website and counting repeated views of a single page"

3b-i) Bug fixes, Downtimes, Content Changes

"We decided to develop for Android since the platform is widely used, is predicted to be the dominant platform in the near future [17], and Android market (now known as Google Play) has a smaller one-time fee of $25 fee for submission of apps."

4a) CONSORT: Eligibility criteria for participants

Users should be able to check web address (http://alsod.iop.kcl.ac.uk) on their phones and/or tablets.

4a-i) Computer / Internet literacy

Users require knowledge of operating a smartphone or tablet to own one.

4a-ii) Open vs. closed, web-based vs. face-to-face assessments:

"Text messages and Blackberry Messenger Messages were sent to a selection of 14 individuals (from 3 continents – North America, Europe and Africa) known to the authors, asking them to view the web address (http://alsod.iop.kcl.ac.uk) on their phones and tablets. "

4a-iii) Information giving during recruitment

No formal documents required.

4b) CONSORT: Settings and locations where the data were collected

"....using Google Analytics tool and our in-house analytic monitoring".

4b-i) Report if outcomes were (self-)assessed through online questionnaires

Online automated tools.

4b-ii) Report how institutional affiliations are displayed

NA

5) CONSORT: Describe the interventions for each group with sufficient details to allow replication, including how and when they were actually administered

5-i) Mention names, credential, affiliations of the developers, sponsors, and owners

"We are especially grateful for the long-standing and continued funding of this project from the ALS Association and the MND Association of Great Britain and Northern Ireland. We also thank ALS Canada, MNDA Iceland and the ALS Therapy Alliance for support. The research leading to these results has received funding from the European Community's Health Seventh Framework Programme FP7/2007-2013 under grant agreement number 259867. We thank the NIHR specialist Biomedical Research Centre for Mental Health and the Biomedical Research Unit for Dementia at the South London and Maudsley NHS Foundation Trust (SLaM) and the Institute of Psychiatry, King's College London. Aleks Radunovic, Nigel Leigh, and Ian Gowrie originally conceived ALSoD. ALSoD is a joint project of the World Federation of Neurology (WFN) and European Network for the Cure ALS (ENCALS)."

5-ii) Describe the history/development process

"The Google Analytics tool was configured in August 2012 and so we based our data analysis on the three months (August, September and October). These first 3 months represented the period from when the Google Analytics tool was implemented to the point where we started the development of the mobile website (which will be referred to as the pre-mobile website period). The post-mobile website period is the second 3 months from November 2012 to January 2013 where the mobile website has been fully developed and an app implemented. In the pre-mobile website period, Pageviews (which is the total number of pages viewed) were analysed to discover the commonly visited pages on the website and counting repeated views of a single page."

5-iii) Revisions and updating

"We chose access to a mobile website for several reasons. First, because mobile websites are immediately accessible to users through a browser, more compatible across devices, have easier content update, are faster to find on search engines, make it easier to share content via a link, have a longer lifecycle on a user's device, are easily convertible to an app and are more cost-effective [13, 30], we used this approach. This approach freezes the interoperability between the mobile-friendly webpages and the Android app file thereby making it easy for developers to replicate the method. Second, the database is regularly updated with data which would require the release of weekly updates to an app if the website was not the primary content holder. Third, although third-party automated app development tools exist, [31, 32] it was simple for us to convert the mobile website into an Android app using WebView object."

5-iv) Quality assurance methods

"We also tested and manipulated the .apk file on a real android phone before submitting to Google Play."

5-v) Ensure replicability by publishing the source code, and/or providing screenshots/screen-capture video, and/or providing flowcharts of the algorithms used

Screenshots and source code for detecting mobile devices is included.

5-vi) Digital preservation

"Once the web address (http://alsod.iop.kcl.ac.uk) is accessed on the browser of any device, the website automatically detects the device type. For example, a mobile phone is redirected to the mobile version pages to fit the smaller screen while desktop or laptop computers display the fuller version. "

5-vii) Access

"The ALS Online Database, (ALSoD) is a freely available website ..."

5-viii) Mode of delivery, features/functionalities/components of the intervention and comparator, and the theoretical framework

"...., we optimized the content layout of the screen, reduced image sizes, and summarized available information. We used the .NET framework mobile detection property which returns a true value if the browser is a recognized mobile device and redirects users automatically. "

5-ix) Describe use parameters

NA

5-x) Clarify the level of human involvement

Apart from the feedback required during the development stage of the mobile website and app, no other human involvement is required after completion.

5-xi) Report any prompts/reminders used

"Text messages and Blackberry Messenger Messages were sent to a selection of 14 individuals..."

5-xii) Describe any co-interventions (incl. training/support)

No training required for users to use the website.

6a) CONSORT: Completely defined pre-specified primary and secondary outcome measures, including how and when they were assessed

NA

6a-i) Online questionnaires: describe if they were validated for online use and apply CHERRIES items to describe how the questionnaires were designed/deployed

No questionnaire was administered.

6a-ii) Describe whether and how "use" (including intensity of use/dosage) was defined/measured/monitored

Users accessed the website on their browsers and went through every webpage to view how it displays on their devices.

6a-iii) Describe whether, how, and when qualitative feedback from participants was obtained

Where the webpages fit their small smartphone screens with minimum or no horizontal scrolls, they sent emails or gave verbal feedback or clicked on the 'recommend' button on the website.

6b) CONSORT: Any changes to trial outcomes after the trial commenced, with reasons

"After the creation, testing and publicity of the app, we received feedback from users about caching for offline viewing [26-28] which would enable users continue work to an extent, page loading icon when connecting, making users aware of cookies policy, using option menu button [29] to display analysis webpages (interaction.aspx, credibility.aspx, analysis.aspx) and creating a link to allow users switch from mobile view to desktop view as this would be useful on tablets like iPad. We were able to make changes on the app based on the feedback except for the offline viewing which could only be possible if the app was not dependent on internet access."

7a) CONSORT: How sample size was determined
7a-i) Describe whether and how expected attrition was taken into account when calculating the sample size
Only 14 individuals were selected. The awareness created on the website boosted the response from the research community thereby making 35 people recommend the website so far.
7b) CONSORT: When applicable, explanation of any interim analyses and stopping guidelines
NA
8a) CONSORT: Method used to generate the random allocation sequence
NA
8b) CONSORT: Type of randomisation; details of any restriction (such as blocking and block size)
NA
9) CONSORT: Mechanism used to implement the random allocation sequence (such as sequentially numbered containers), describing any steps taken to conceal the sequence until interventions were assigned
NA
10) CONSORT: Who generated the random allocation sequence, who enrolled participants, and who assigned participants to interventions
NA
11a) CONSORT: Blinding - If done, who was blinded after assignment to interventions (for example, participants, care providers, those assessing outcomes) and how
11a-i) Specify who was blinded, and who wasn't
NA
11a-ii) Discuss e.g., whether participants knew which intervention was the "intervention of interest" and which one was the "comparator"
NA
11b) CONSORT: If relevant, description of the similarity of interventions
NA
12a) CONSORT: Statistical methods used to compare groups for primary and secondary outcomes
NA
12a-i) Imputation techniques to deal with attrition / missing values
NA
12b) CONSORT: Methods for additional analyses, such as subgroup analyses and adjusted analyses
NA

RESULTS

13a) CONSORT: For each group, the numbers of participants who were randomly assigned, received intended treatment, and were analysed for the primary outcome
NA
13b) CONSORT: For each group, losses and exclusions after randomisation, together with reasons
NA
13b-i) Attrition diagram
NA
14a) CONSORT: Dates defining the periods of recruitment and follow-up
NA
14a-i) Indicate if critical "secular events" fell into the study period
NA
14b) CONSORT: Why the trial ended or was stopped (early)
NA
15) CONSORT: A table showing baseline demographic and clinical characteristics for each group
NA
15-i) Report demographics associated with digital divide issues
NA
16a) CONSORT: For each group, number of participants (denominator) included in each analysis and whether the analysis was by original assigned groups
16-i) Report multiple "denominators" and provide definitions
NA
16-ii) Primary analysis should be intent-to-treat
NA
17a) CONSORT: For each primary and secondary outcome, results for each group, and the estimated effect size and its precision (such as 95% confidence interval)
NA
17a-i) Presentation of process outcomes such as metrics of use and intensity of use
NA
17b) CONSORT: For binary outcomes, presentation of both absolute and relative effect sizes is recommended
NA
18) CONSORT: Results of any other analyses performed, including subgroup analyses and adjusted analyses, distinguishing pre-specified from exploratory
NA
18-i) Subgroup analysis of comparing only users
NA
19) CONSORT: All important harms or unintended effects in each group
NA
19-i) Include privacy breaches, technical problems
NA
19-ii) Include qualitative feedback from participants or observations from staff/researchers
NA

DISCUSSION

20) CONSORT: Trial limitations, addressing sources of potential bias, imprecision, multiplicity of analyses
20-i) Typical limitations in ehealth trials
NA
21) CONSORT: Generalisability (external validity, applicability) of the trial findings
21-i) Generalizability to other populations
"...14 individuals (from 3 continents – North America, Europe and Africa) known to the authors, asking them to view the web address (http://alsod.iop.kcl.ac.uk) on their phones and tablets."
21-ii) Discuss if there were elements in the RCT that would be different in a routine application setting
NA
22) CONSORT: Interpretation consistent with results, balancing benefits and harms, and considering other relevant evidence
22-i) Restate study questions and summarize the answers suggested by the data, starting with primary outcomes and process outcomes (use)
NA
22-ii) Highlight unanswered new questions, suggest future research
"Our future work shall be concentrating on further integration with other databases for a wider interpretation of genetic data in ALS across the globe."

Other information

23) CONSORT: Registration number and name of trial registry
NA
24) CONSORT: Where the full trial protocol can be accessed, if available
NA
25) CONSORT: Sources of funding and other support (such as supply of drugs), role of funders
"We are especially grateful for the long-standing and continued funding of this project from the ALS Association and the MND Association of Great Britain and Northern Ireland. We also thank ALS Canada, MNDA Iceland and the ALS Therapy Alliance for support. The research leading to these results has received funding from the European Community's Health Seventh Framework Programme FP7/2007-2013 under grant agreement number 259867. We thank the NIHR specialist Biomedical Research Centre for Mental Health and the Biomedical Research Unit for Dementia at the South London and Maudsley NHS Foundation Trust (SLaM) and the Institute of Psychiatry, King's College London. Aleks Radunovic, Nigel Leigh, and Ian Gowrie originally conceived ALSoD. ALSoD is a joint project of the World Federation of Neurology (WFN) and European Network for the Cure ALS (ENCALS)."
X26-i) Comment on ethics committee approval
NA
x26-ii) Outline informed consent procedures
NA
X26-iii) Safety and security procedures
NA
X27-i) State the relation of the study team towards the system being evaluated
No conflict of interest
